# 

**Published:** 1907-08-03

**Authors:** 


### BOOKS RECEIVED.

CASSELL AND CO.

"Gout: its Pathology, Forms, Diagnosis, and Treatment." 3rd edition. By A. P. Luff, M.D.

"Bilharziosis." By F. C. Madden, M.D.

W. HEINEMANN.

"The Efficient Life." By Luther H. Gulick, M.D.

THE CENTRAL PUBLISHING CO.

"Health for Children." By T. E. Saunt, M.R.C.S.

MACMILLAN AND CO.

"Alcohol and the Human Body." By Sir Victor Horsley, F.R.S., and Mary D. Sturge, M.D.

WYMAN AND SONS, LTD.

"History of the Wine Trade in England." Vol. I. By A. L. Simon.

FUNK AND WAGNALL CO.

"Home Gymnastics on Ling's System." By Professor Anders Wide, M.D.

H. K. LEWIS.

"What to do in Cases of Poisoning." 10th edition. By W. Murrell, M.D.

### Resident Medical Officers' Department.

### NOTES, QUESTIONS, AND COMMENTS.

The editor of the Resident Medical Officers' Department of The Hospital invites contributions to this column both from resident medical officers and from other members of the profession. Short articles, notes, queries, or suggestions bearing upon this branch of medical practice will always receive careful consideration, and those that are suitable will be published in due course. Correspondence, short and to the point, is particularly invited, as by this means the value of the R.M.O. Department will be greatly increased.

Articles, whether in the form of letters to the editor or otherwise, should in no case exceed 600 words in length, and should, if possible, be shorter than this. Postcards with short notes, questions, or notifications of recent hospital appointments will be welcomed.

All communications for this column must be guaranteed by the name of the writer, which will not be published unless he expressly wishes it, and should bear the words "R.M.O. Department" on the envelope or the address side of the postcard.

Items of news from the resident officers' quarters of the principal hospitals, infirmaries, and asylums throughout the kingdom are invited, and, when of sufficient general interest, will be published.

The editor will also be pleased to receive and reply to confidential communications from resident medical officers and to consider any suggestions that may be made for the improvement of this department of The Hospital.

Brief notes and comments on new methods of diagnosis and treatment which are under trial in hospitals and are likely to be of practical use to readers will be especially welcome; and we shall be glad to receive questions or comments bearing on these subjects both from general practitioners and from R.M. officers.

### NOTICES AND ANSWERS TO CORRESPONDENCE.

All MSS., letters, books for review, and other matters intended for the Editor, should be addressed to:—

The Editor,
The Hospital Building,
28 and 29 Southampton Street,
Strand, London, W.C.

### Contributions.

Contributions should be written, or preferably typed, on one side of the paper only, and all articles sent in are accepted upon the distinct understanding that they are forwarded to The Hospital only.

### Correspondence.

Correspondence on all subjects is invited, but no communication can be entertained if the name and address of the correspondent are not given as a guarantee of good faith, but not necessarily for publication. All correspondents should write on one side of the paper only.

### Books for Review.

Publishers are particularly requested to send advance proofs of any new books of importance, whenever possible, as the Editor has made arrangements to publish immediate reviews, and on a new plan.

### BUSINESS NOTICES.

Letters relating to the Publishing, Sale and Advertisement Departments must be addressed to the Manager (*not to the Editor*):—

The Manager,
The Hospital Building,
28 and 29 Southampton Street,
Strand, London, W.C.

### Annual Subscriptions.

The rate of annual subscriptions to The Hospital, either direct from the Office or through agents, is:—

For the United Kingdom ... ... 13s. a year
To the Colonies and Abroad ... 17s. a year
inclusive of postage in each case.

### Subscriptions.

Subscriptions are payable in advance. Cheques and Postal Orders should be made payable to:—

The Scientific Press, Ltd., and should be crossed: London and County Banking Co., Ltd.

### Cases for Binding.

Cases for binding half-yearly volumes of The Hospital may be obtained from the Manager, price 2s., post free. The Index to Vol. xli. is now ready, price 3½d., post free. The monthly part for July is also ready, price 1s., by post 1s. 3d.

